# Artificial intelligence for white matter hyperintensity segmentation: performance, determinants, and clinical implications

**DOI:** 10.3389/fnagi.2026.1930675

**Published:** 2026-09-16

**Authors:** Jinhua Hu, Zhihao Zhang, Peng Lei, Jupeng Zhang, Xiqi Zhu, Baosheng Li

**Affiliations:** 1 Department of Radiology, Affiliated Hospital of Youjiang Medical University for Nationalities, Baise, China; 2 School of Medical Laboratory Science, Affiliated Hospital of Youjiang Medical University for Nationalities, Baise, China; 3 Guangxi Key Laboratory of Artificial Intelligence for Genetic Diseases of Long-dwelling Nationalities, Baise, Guangxi, China

**Keywords:** artificial intelligence, deep learning, meta-analysis, systematic review, U-Net, white matter hyperintensities

## Abstract

**Objective:**

White matter hyperintensities (WMH) are key neuroimaging markers of cerebral small vessel disease, associated with cognitive decline and stroke risk. Accurate quantification is essential yet manual segmentation is time-consuming and variable. We systematically evaluated AI performance for automated WMH segmentation across disease contexts and identified factors influencing model performance.

**Methods:**

Following PRISMA guidelines, we searched PubMed, Embase, Web of Science, Scopus, and IEEE Xplore from database inception to February 3, 2026 (PROSPERO: CRD420261355769). Studies employing AI-based WMH segmentation and reporting Dice similarity coefficient (DSC) were included. A random-effects meta-analysis was conducted, along with subgroup analyses, meta-regression, sensitivity analyses, and publication bias assessment.

**Results:**

26 studies comprising 4,288 participants contributed to quantitative syntheses: 13 evaluated WMH segmentation in general cohorts, and 13 evaluated AD or CSVD-specific cohorts. In the general cohort meta-analysis, the pooled DSC was 0.78 (95% CI, 0.74–0.82). Disease-specific pooled DSC estimates were 0.75 for AD and 0.78 for CSVD. Exploratory meta-regression suggested an association between scanning parameters and DSC (*p* = 0.042), whereas magnetic field strength showed a borderline association (*p* = 0.052).

**Conclusion:**

AI demonstrates promising performance for automated WMH segmentation, with U-Net–based architectures showing superior accuracy. However, substantial heterogeneity highlights the need for standardized imaging protocols and robust external validation. Future multicenter studies are essential to improve model generalizability and facilitate clinical translation.

## 1 Introduction

White matter hyperintensities (WMH) represent the most common neuroimaging manifestation of cerebral small vessel disease (CSVD) and are highly prevalent in the aging population as well as in patients with neurodegenerative disorders (Cheng et al., 2025; Qiu et al., 2025). On T2-weighted and T2-FLAIR magnetic resonance imaging (MRI), WMHs typically appear as hyperintense lesions in the periventricular and deep white matter (Duering et al., 2023; Wardlaw et al., 2013). Accurate quantification of WMH burden is essential for understanding their clinical significance and monitoring disease progression. Previous studies have demonstrated that WMHs are associated with cognitive decline, gait disturbances, dementia, and an increased risk of recurrent stroke (Jansma et al., 2024; Iveson et al., 2026; Johnsen et al., 2025; Whitman et al., 2001). As global population aging accelerates, precise and automated WMH quantification has become increasingly important for both neuroimaging research and clinical practice.

Manual delineation performed by expert raters remains the reference standard for WMH quantification. However, this approach is time-consuming, labor-intensive, and subject to substantial intra- and inter-observer variability, limiting its applicability in large-scale studies. Early semi-automated techniques, such as threshold-based or region-growing methods, have improved efficiency to some extent (Ramirez et al., 2011). Nevertheless, their robustness and cross-scanner generalizability remain limited due to the indistinct boundaries and heterogeneous characteristics of WMHs.

In recent years, artificial intelligence (AI), particularly deep learning approaches based on convolutional neural networks (CNNs), has demonstrated considerable potential in medical image segmentation (Ilesanmi et al., 2024). Among these, U-Net and its variants have become the dominant architectures owing to their encoder–decoder design and skip connections, which facilitate multi-scale feature fusion and improve segmentation accuracy (Zhang et al., 2020). More recently, advanced frameworks such as nnU-Net and Transformer-based models have further improved segmentation performance (Qin et al., 2026). However, their generalizability and clinical applicability remain to be fully validated.

Despite the rapid growth of AI-based WMH segmentation studies, several important issues remain unresolved. First, systematic comparisons across different algorithms are limited, as most studies focus on optimizing individual models. Second, WMH characteristics vary across disease contexts, such as Alzheimer’s disease (AD) and CSVD, and the stability of AI models under different pathological conditions remains unclear. Third, reported Dice similarity coefficient (DSC) values vary widely (0.52–0.92), suggesting that segmentation performance may be influenced by multiple factors, including dataset heterogeneity, imaging parameters, and algorithm architecture (Kervadec et al., 2021). Finally, a comprehensive meta-analytic evaluation of key moderating variables—such as field strength, external validation, and multicenter data—remains lacking.

Therefore, this systematic review and meta-analysis aimed to address these gaps through four objectives: (1) to compare the performance of different AI algorithms for WMH segmentation; (2) to evaluate the influence of disease context on segmentation accuracy; (3) to estimate the pooled performance of AI-based WMH segmentation using DSC; and (4) to explore potential moderators, including imaging parameters and validation strategies, using meta-regression analysis. Ultimately, this study aims to provide evidence-based guidance for optimizing automated WMH segmentation models and facilitating their clinical translation.

## 2 Materials and methods

### 2.1 Search strategy and registration

This systematic review and meta-analysis was designed and reported in strict accordance with the PRISMA (the Preferred Reporting Items for Systematic Reviews and Meta-Analyses) guidelines (McInnes et al., 2018). The study protocol was registered with the International Prospective Registry of Systematic Reviews (PROSPERO) prior to data extraction (registration number CRD420261355769).

A systematic search was conducted across five electronic databases: PubMed, IEEE Xplore, Scopus, Web of Science, and Embase. The search covered the period from database inception to February 3, 2026. The search strategy was built around three core concepts—namely, “white matter hyperintensity,” “artificial intelligence,” and “segmentation”—and employed a combination of Medical Subject Headings (MeSH) terms and free-text keywords. The detailed search strategies adopted for each database are provided in the Supplementary materials. Two researchers (J.H. and Z.Z.) independently reviewed the search results and cross-checked their findings to ensure that retrieval was complete.

### 2.2 Inclusion and exclusion criteria

This study established predefined inclusion criteria based on the PICOS principle: study participants were adult subjects undergoing brain MRI examinations, including healthy controls and individuals with AD, or CSVD; the intervention or testing method involved the use of AI technology (including but not limited to deep learning and machine learning) for the segmentation of white matter hyperintensities; The reference standard included manual segmentation by experts or validated semi-automated methods; the outcome measure is the DSC; and the study type is original research, including prospective, retrospective, and cross-sectional studies.

Exclusion criteria include: non-original studies (e.g., reviews, conference abstracts, commentaries, editorials, case reports, and guidelines); non-human studies; studies involving participants under 18 years of age; studies not using AI-based segmentation methods; studies that did not report or could not calculate the DSC; non-English language literature; and duplicate publications (only the study with the most complete data or the most recent publication date will be included).

### 2.3 Study selection and data extraction

After importing retrieved records into EndNote and removing duplicates, two researchers (J.H. and Z.Z.) independently performed a two-stage screening process: an initial assessment based on titles and abstracts, followed by a full-text review of potentially eligible articles. Disagreements regarding eligibility were resolved by consensus or, when necessary, by consultation with a third researcher (X.Z.). The screening process is summarized in the PRISMA flowchart.

The same two researchers independently extracted data using a standardized form that captured: study characteristics (first author, publication year, country/region, study design, sample size, participant age, and sex); MRI parameters (scanner manufacturer, magnetic field strength, and sequence type); AI model characteristics (algorithm type, use of multicenter data, use of external validation, and development environment); and performance metrics (i.e., mean and standard deviation of the DSC). Extracted data were subsequently cross-checked for accuracy, and corresponding authors were contacted to request additional information where necessary. In addition to study and model characteristics, we extracted information on reference-label construction, including the annotation method, number and expertise of raters where reported, use of consensus or adjudication procedures, semi-automated processing followed by manual editing, and the availability of inter-rater reliability data. Reference-label construction was classified as manual annotation (single-rater or multi-rater consensus/adjudication), semi-automated segmentation followed by manual editing, or insufficiently reported. No included study was identified as using an automated algorithm alone as the reference standard. Detailed study-level information is presented in Supplementary Table S2.

### 2.4 Quality assessment

Two researchers (J.H. and Z.Z.) independently assessed study quality using the QUADAS-2 tool for diagnostic accuracy studies. The QUADAS-2 framework comprises—patient selection, index test, reference standard, and flow and timing—each of which is evaluated for risk of bias as well as concerns regarding applicability. Each study received a classification of low, high, or unclear risk; a summary of the assessment is provided in Supplementary Table S1. Any disagreements were resolved by consensus or by consultation with a third researcher (X.Z.).

### 2.5 Statistical analysis

This study used the DSC as the primary outcome measure and employed a random-effects model using the DerSimonian–Laird method for meta-analysis to account for the expected heterogeneity among studies. For each study, the 95% confidence interval (CI) of the pooled DSC was calculated from the reported mean and standard deviation, while heterogeneity across studies was assessed with the *I*^2^ statistic (where *I*^2^ > 50% suggests substantial heterogeneity and *I*^2^ > 75% indicates considerable heterogeneity).

To explore the sources of heterogeneity, a subgroup analysis was conducted. Studies were grouped by algorithm type into three categories: those employing U-Net architectures and their variants, those utilizing other deep learning approaches, and those relying on traditional convolutional neural networks. Pooled DSC estimates were then calculated for each subgroup. In parallel, we conducted univariate meta-regression to examine the association between potential covariates and reported DSC values. Covariates considered included algorithm type, scanning parameters, use of external validation, field strength, availability of multicenter data, and country. Because reference-label categories were sparse and reporting was incomplete, a reliable confirmatory subgroup analysis or meta-regression by reference-standard type was not feasible. We therefore conducted only an exploratory univariable analysis of reference-standard type, summarized reference-label construction descriptively, and interpreted any association cautiously.

A leave-one-out sensitivity analysis was undertaken to evaluate the stability of the pooled estimates. Publication bias was assessed using the Begg rank correlation test, the Egger linear regression test, and visual inspection of funnel plots. A *p*-value < 0.10 on Egger’s test or <0.05 on Begg’ test was taken to suggest potential publication bias. All statistical analyses were conducted using Stata (version 17.0 or later), and a two-tailed *p* < 0.05 was considered statistically significant.

## 3 Results

### 3.1 Study selection

The database searches identified 2,594 records. After the removal of 647 duplicates, 1,947 records underwent title and abstract screening, of which 1,778 were excluded. The full texts of 169 reports were sought for retrieval, and 11 reports could not be obtained. Consequently, 158 full-text reports were assessed for eligibility. Of these, 132 were excluded, including 17 animal studies, 73 reviews, letters, guidelines, editorials, or conference abstracts, 14 studies not specifically addressing WMH segmentation, and 28 studies involving infants or children. Ultimately, 26 studies were included in the systematic review and meta-analysis (Figure 1; Zhao et al., 2021, 2025; Zhang et al., 2018, 2021; Wu et al., 2019, 2026; Viteri et al., 2022; Tsuchida et al., 2024; Sundaresan et al., 2022; Rieu et al., 2021; Rachmadi et al., 2018; Mojiri Forooshani et al., 2022; Lo et al., 2024; Hotz et al., 2022; Shan et al., 2021; Li et al., 2022; Jeong et al., 2025; Huang et al., 2023; He et al., 2025; Fiford et al., 2020; Eldianto et al., 2023; Vanderbecq et al., 2020; Røvang et al., 2023; Pham et al., 2021; Park et al., 2023; Liang et al., 2021).

**Figure 1:**
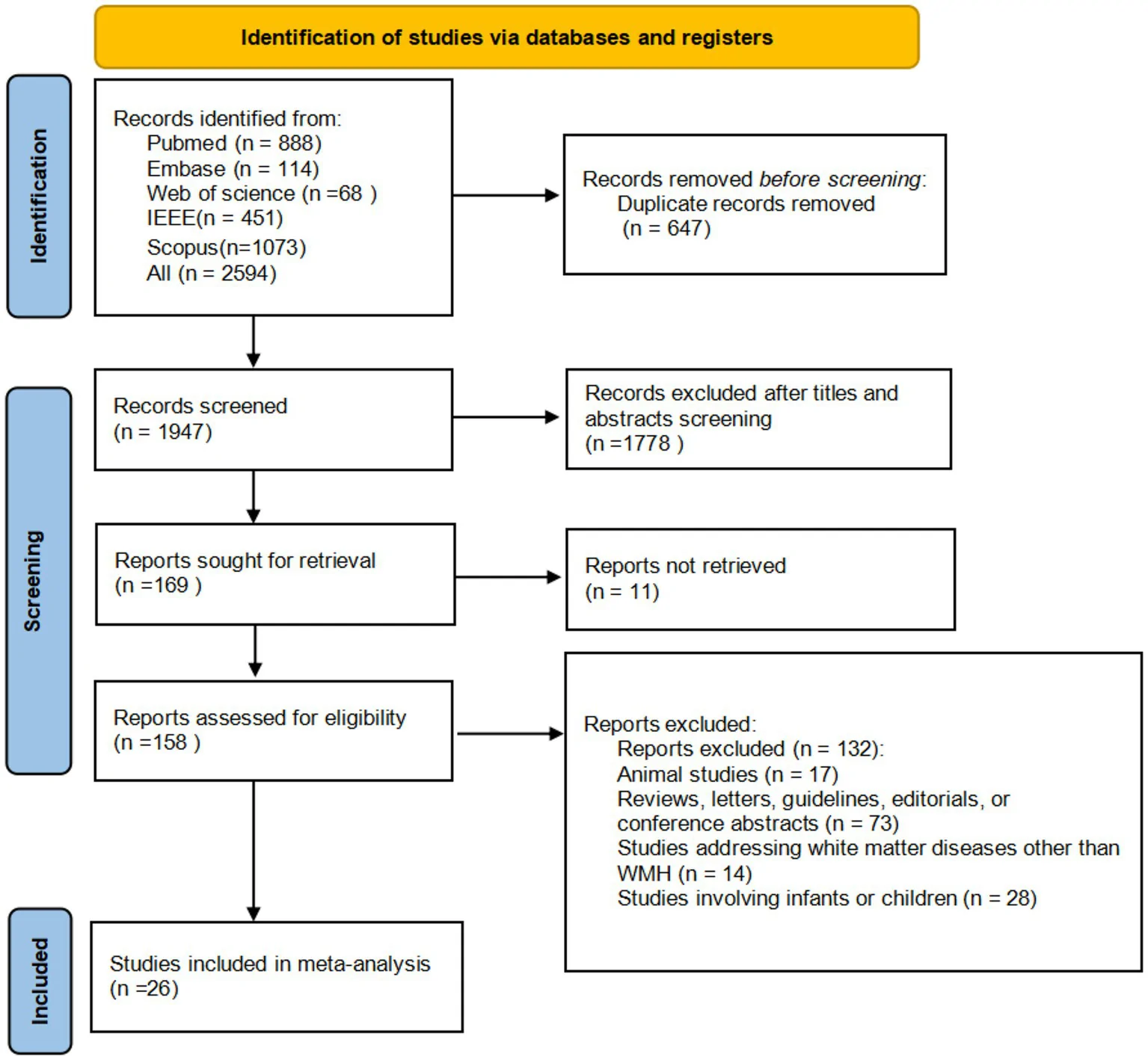
PRISMA flowchart of study selection for the systematic review and meta-analysis. The flowchart outlines the number of records identified, screened, excluded, and finally included in the systematic review and meta-analysis, following PRISMA guidelines.

### 3.2 Basic characteristics of included studies

The 26 included studies were published between 2017 and 2026 and originated from several countries, predominantly China (*n* = 10), followed by the United States, South Korea, France, and Canada. These studies enrolled a total of 4,288 participants, including healthy controls, individuals with WMH, and patients diagnosed with AD or CSVD. The MRI protocols primarily included T1 and T2-FLAIR sequences, and the field strengths were predominantly 1.5 T or 3.0 T. The U-Net architecture and its derivatives were the most commonly employed AI segmentation frameworks, while a subset of studies reported the use of multicenter data and/or performance of external validation. Comprehensive characteristics of the included studies are provided in Table 1, and details regarding the AI models are presented in Table 2.

**Table 1:** Characteristics of the selected studies.

First author	Year	Country/Region	Study design	Sample size	Age (years)	Sex (M/F)	Scanner	MRI sequences
Zhao	2021	China	NR	NR	NR	NR	NR	T1
Zhang	2018	China	NR	NR	NR	NR	1.5, 3.0MR	T1
Zhang(b)	2021	China	NR	NR	NR	NR	NR	T1, T2-FLAIR
WU	2017	China	NR	NR	NR	NR	NR	T1, FLAIR
WU(b)	2026	United States	Retrospective	NR	NR	NR	1.5 T, 3 T (Siemens, GE, Philips)	T1, T2-FLAIR
Viteri	2022	Ecuador	NR	NR	NR	NR	3 T (Philips, Siemens, GE)	T1, FLAIR
Tsuchida	2023	France	NR	NR	NR	NR	3 T (Philips, Siemens, GE)	T1, FLAIR
Sundaresan	2022	United Kingdom	NR	NR	NR	NR	NR	NR
Rieu	2021	Korea	NR	NR	NR	NR	NR	FLAIR
Rachmadi	2018	United Kingdom	NR	NR	NR	NR	NR	T1
Forooshani	2021	Canada	NR	NR	NR	NR	NR	T1, T2-FLAIR
Lo	2024	Taiwan, China	NR	NR	NR	NR	NR	T2-FLAIR
Hotz	2022	Switzerland	NR	NR	NR	NR	3 T (Philips)	T1
Zhao(b)	2025	China	cohort	163	64.8 ± 7.4 years	70/35	3 T (GE)	T1, T2-FLAIR
Shan	2021	China	Retrospective	Internal test: 160 External test: 90	Median 54	Development: 653/503	1.5 T, 3 T	T2-FLAIR
Li	2022	China/Australia	Retrospective	MWC: 60 CNSR: 60	NR	37/23	3 T (Philips, Siemens, GE)	T1, T2-FLAIR
Jeong	2025	South Korea	Retrospective	1974	71.1 ± 9.7 years	685/1,109	NR	T2-FLAIR
Huang	2022	China	Retrospective	Total: 455 HKU-SVD: 360 HKU-MS: 20 MICCAI-WMH: 60 ADNI-CN: 15	NR	NR	3 T (GE, Philips, Siemens)	T1, T2-FLAIR
He	2025	China	Retrospective	Total: 366 VCI: 126 WMC: 170 VRF: 70	45–75	NR	3 T (Siemens, GE, Philips) 1.5 T (GE)	T1, T2-FLAIR
Fiford	2020	United Kingdom	NR	60 (30 controls, 30 AD)	71.0–74.9	Controls: 83/97 AD: 85/66	3 T (GE, Philips)	T1, T2-FLAIR
Eldianto	2023	Indonesia	NR	ADNI: 60 MRI volumes (20 patients × 3 time points)	NR	NR	3 T GE	T1, T2-FLAIR
Vanderbecq	2020	France	NR	ADNI: 147	ADNI: 58–90	ADNI: 66/81	3 T GE	T1 FLAIR
Røvang	2023	Norway	cohort	Internal: 441 External: 29	64.9 ± 9.3	194/247	3 T (Siemens, Philips) 3 T, 1.5 T GE	T1 FLAIR
Pham	2021	Vietnam	NR	NR	NR	NR	NR	T1
Park	2022	South Korea	NR	Training: 301 CN Test: 480 (276 AD, 204 CN)	AD:76.63 ± 5.84 CN:77.51 ± 5.71	Training: 137/164 AD: 144/132 CN: 122/82	NR	T1
Liang	2021	China	NR	ADNI: 285 (94 CN, 80 AD)	75.10 ± 7.66	41/39	3 T (Philips, Siemens, GE)	T1, T2-FLAIR

NR, not reported; AD, Alzheimer’s disease; MCI, mild cognitive impairment; CN, cognitively normal; VCI, vascular cognitive impairment; WMC, white matter changes; VRF, vascular risk factors; FLAIR, fluid-attenuated inversion recovery. Sample sizes are presented as total number of participants unless otherwise specified. Age is shown as mean ± standard deviation or range. MRI sequences include T1-weighted, T2-FLAIR, and FLAIR. Studies span multiple countries and scanner types.

**Table 2:** The characteristics of artificial intelligence selected for the research.

Author	Year	External validation	Multicenter	Algorithms	Utilization software
Zhao	2021	No	Yes	3D U-Net	Python, Keras
Zhang	2018	No	Yes	U-Net	Keras, TensorFlow, Ubuntu
Zhang(b)	2021	No	Yes	GLM-Net	Keras, NVIDIA RTX 2080 GPU
WU	2017	No	Yes	SC U-Net	Keras (TensorFlow backend), Ubuntu 16.04, GTX
WU(b)	2026	Yes	No	nnU-Net	ITK-SNAP, ANTs, FSL, PyTorch
Viteri	2022	No	Yes	Swin U-Net, U-Net	Python 3.7, PyTorch 1.x, ANTs, scipy
Tsuchida	2023	Yes	Yes	3D U-Net	TensorFlow 2.7 (Keras backend), Python 3.7
Sundaresan	2022	No	Yes	2D U-Net	PyTorch 1.2.0
Rieu	2021	No	Yes	U-Net, U-Net++, HighRes3DNet	PyTorch, TorchIO, FreeSurfer, SimpleITK
Rachmadi	2018	No	Yes	CNN	FSL (FLIRT, FAST), optiBET, Analyze™, Mango, Python
Forooshani	2021	Yes	Yes	CNN	PyTorch, FSL (BIANCA), DeepMedic, Hypermapp3r
Lo	2024	No	No	U-Net	NR
Hotz	2022	No	No	KNN	FreeSurfer v6.0.1, UBO Detector, FSL (BIANCA), fMRIPrep, ANTs, MATLAB
Zhao(b)	2025	Yes	No	U-Net, Res-Net, DeepLabV3	ITK-SNAP, SimpleITK, Nibabel, PyTorch, Python
Shan	2021	Yes	Yes	U-Net	NR
Li	2022	Yes	Yes	U-Net, SE-Net	NR
Jeong	2025	Yes	No	U-Net	ITK-SNAP
Huang	2022	Yes	Yes	V-Net	SPM12, Elastix 4.8, FSL (BET, FAST), ITK-SNAP 3.8.0, Wolfram Mathematica 12.0
He	2025	Yes	Yes	TrUE-Net	MRIcroGL, ANTs, Deep-Brain, FSL (FLIRT, FAST), ITK-SNAP
Fiford	2020	No	Yes	BaMoS (Bayesian Model Selection)	NiftyReg, NiftyMIDAS, BMAPS
Eldianto	2023	Yes	Yes	U-Net	Python, TensorFlow 2.5.0, keras-unet-collection, Nvidia DGX-1
Vanderbecq	2020	No	Yes	NR	SPM12 (LST toolbox), FSL (BIANCA), ITK-SNAP, MATLAB
Røvang	2023	Yes	Yes	U-Net, nnU-Net	PyTorch, MONAI, NVIDIA NGC, Keras, HD-BET, Mcverter, ANTs, scikit-image
Pham	2021	No	No	U-Net	Python, Keras
Park	2022	Yes	Yes	U-Net	FreeSurfer, Python, Keras
Liang	2021	Yes	Yes	U-Net	FSL (FLIRT, BET), ANTs, Brain Label, Python, R

NR, not reported; CNN, convolutional neural network; KNN, k-nearest neighbors. Algorithms include various U-Net variants and other deep learning architectures. Utilization software lists frameworks and tools used for model development and image processing. External validation indicates whether models were tested on independent datasets.

### 3.3 Quality assessment results

Quality of the included studies was assessed with the QUADAS-2 tool; a summary of the findings is presented in Supplementary Table S1. The assessment encompassed the four QUADAS-2 domains: patient selection, index test, reference standard, and flow and timing. For the majority of studies, risk of bias was deemed low across all domains, and concerns regarding applicability were generally minimal. These findings suggest that the overall methodological quality of the evidence base was high.

### 3.4 Meta-analysis results

#### 3.4.1 Overall segmentation performance

A total of 13 studies contributed data on the DSC for WMH segmentation. The random-effects meta-analysis yielded a pooled DSC estimate of 0.78 (95% confidence interval [CI], 0.74–0.82), accompanied by substantial heterogeneity across studies (*I*^2^ = 98.7%, *p* < 0.001; Figure 2). Individual study DSC estimates ranged from 0.52 to 0.92. Among the studies that included AD patients, eight contributed data, producing a pooled DSC of 0.75 (95% CI, 0.68–0.83) and exhibiting an *I*^2^ of 99.4%. Similarly, five studies provided data on CSVD patients, for whom the pooled DSC was 0.78 (95% CI, 0.72–0.85) with an *I*^2^ value of 99.1% (Figure 3).

**Figure 2:**
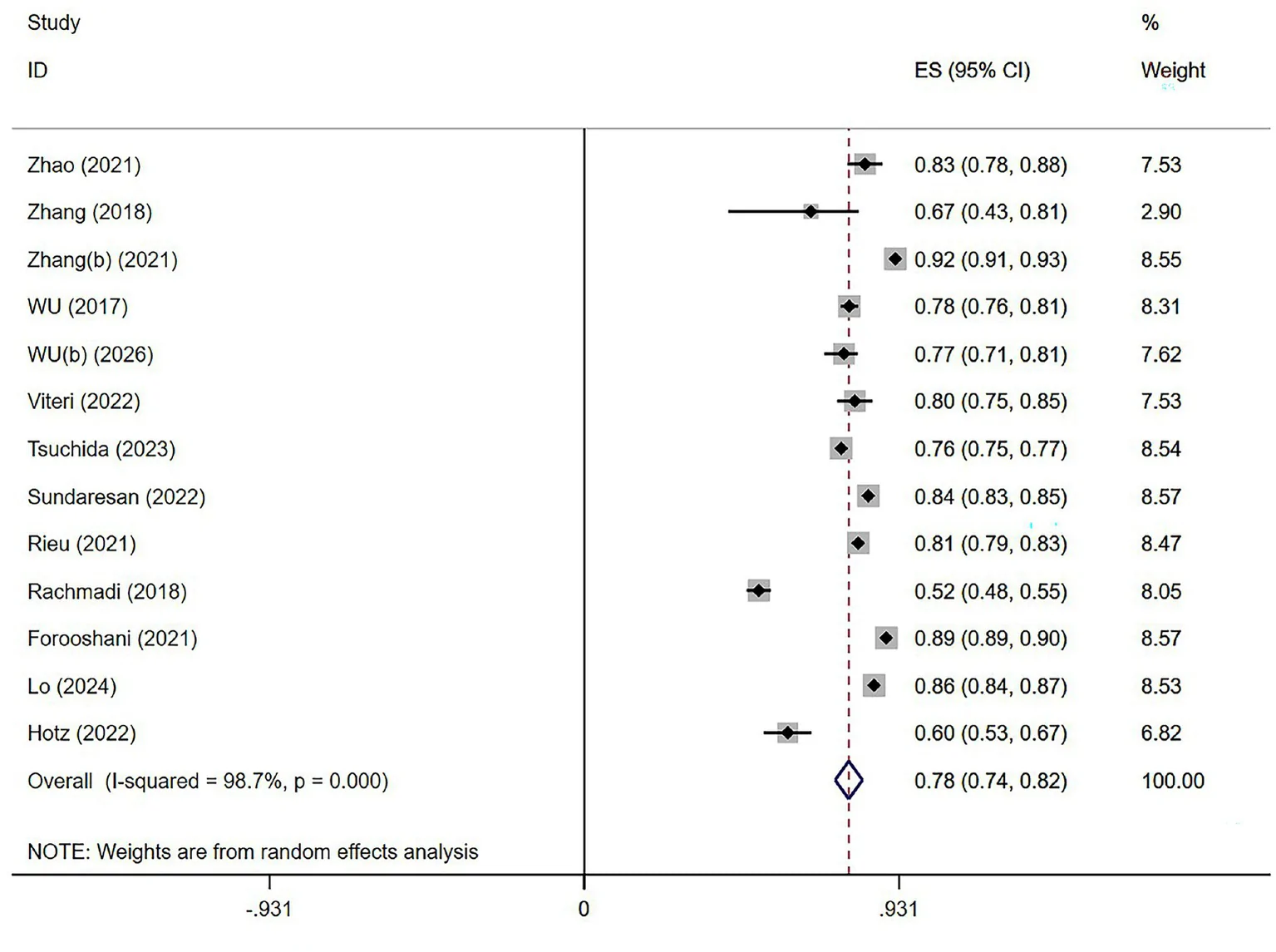
Forest plot of the combined Dice similarity coefficient (DSC) for white matter hyperintensity segmentation. WMH, white matter hyperintensity.

**Figure 3:**
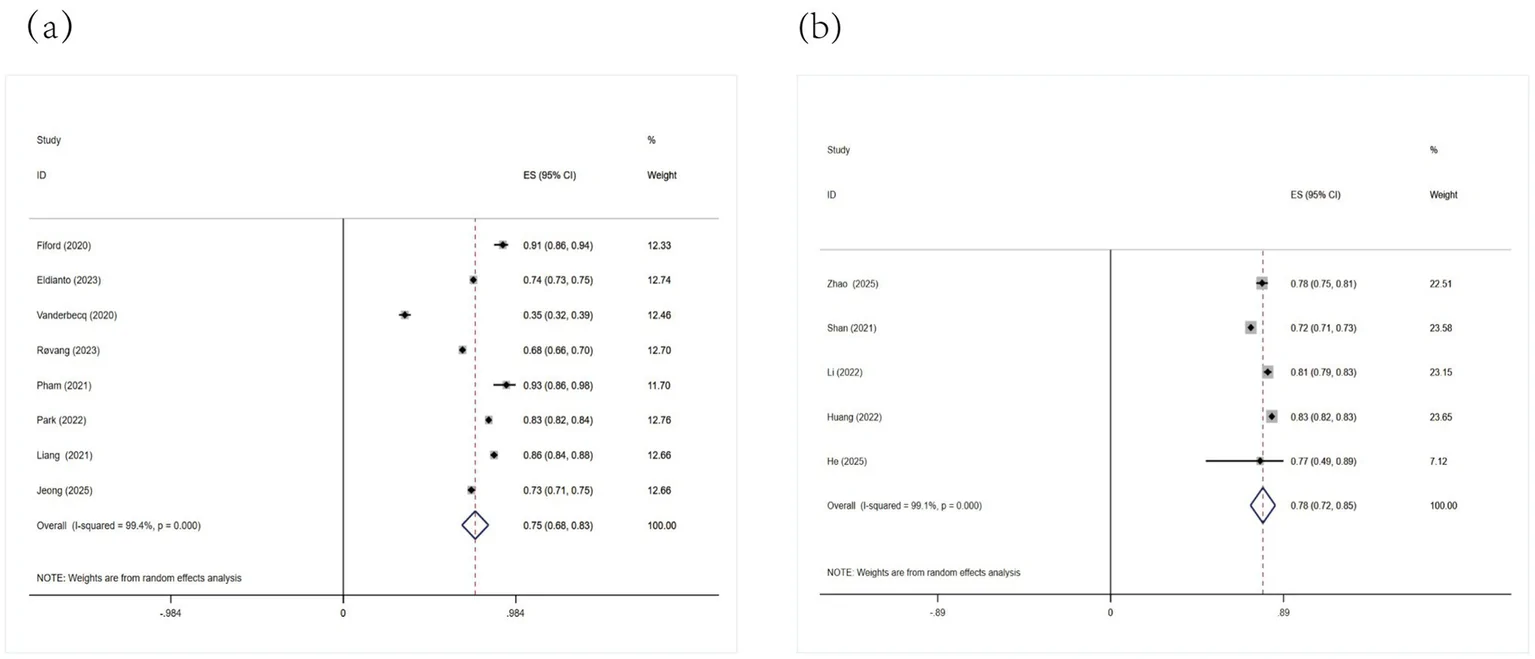
Forest plot of the combined Dice similarity coefficient for white matter hyperintensity segmentation by disease type. Panels **(a,b)** present pooled Dice similarity coefficients for Alzheimer’s disease (AD) and cerebral small vessel disease (CSVD), respectively. Each panel includes individual study estimates and summary effects.

#### 3.4.2 Algorithm subgroup analysis

A subgroup analysis was performed according to algorithm type (Figure 4), which classified studies into three groups: those employing U-Net architectures and their variants, those using other deep learning approaches, and those relying on traditional CNNs. The pooled DSC estimate for studies employing U-Net architectures and their variants was 0.80 (95% CI, 0.76–0.84), compared with 0.76 (95% CI, 0.70–0.82) for those utilizing other deep learning methods and 0.74 (95% CI, 0.67–0.81) for those applying traditional CNNs. Substantial heterogeneity was evident within each subgroup.

**Figure 4:**
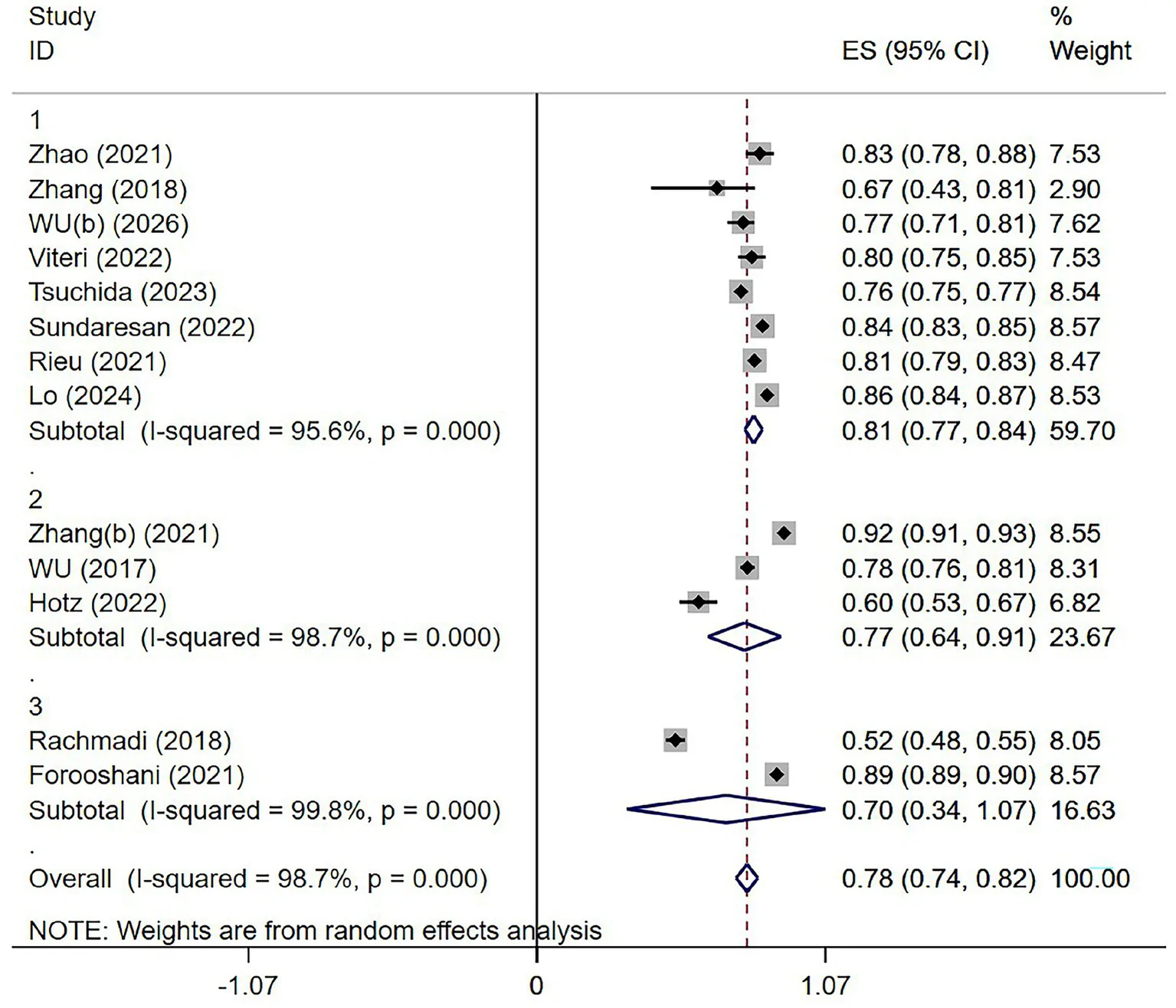
Subgroup analysis (forest plot) of Dice similarity coefficients based on the algorithm type (U-Net, other, CNN). Subgroup analysis compares segmentation performance across algorithm categories: U-Net, other deep learning architectures, and conventional convolutional neural networks (CNNs). Each subgroup shows pooled DSC with 95% confidence intervals.

#### 3.4.3 Meta-regression analysis

Reference-label construction varied across the included studies. Thirteen studies used manual annotation, including single-rater annotation or multi-rater consensus/adjudication; four used semi-automated segmentation followed by manual editing; and nine did not provide sufficient information to determine the construction procedure. No study was identified as using an automated algorithm alone as the reference standard. These data are summarized in Supplementary Table S2.

The findings from the univariate meta-regression analysis are presented in Table 3. In exploratory univariable meta-regression, scanning parameters were associated with reported DSC values (*p* = 0.042), whereas magnetic field strength showed a borderline association (*p* = 0.052). Given the limited number of studies and incomplete reporting of some covariates, these findings should be interpreted cautiously and considered hypothesis-generating rather than definitive. In contrast, no significant associations were detected for algorithm type, external validation, multicenter data, or country (all *p* > 0.05). In an exploratory univariable analysis, reference-standard type classified as manual annotation (single-rater or multi-rater consensus/adjudication), semi-automated segmentation followed by manual editing, or insufficiently reported was not significantly associated with reported DSC values (*p* = 0.245). Given the sparse categories and incomplete reporting, this result should not be interpreted as evidence that reference-standard variability does not contribute to DSC variation or heterogeneity.

**Table 3:** Univariate meta-regression analysis of factors affecting the Dice similarity coefficient for white matter hyperintensity segmentation.

Brain region	Covariate	Coef.	*p*
WMH	Algorithms	−0.1334196	0.853
	Scanning	2.351612	0.042
	External	0.6191766	0.608
	Software	−1.504156	0.353
	Field strength	1.481695	0.052
	Multicenter	2.851817	0.189
	Country	0.1755006	0.522
	Reference standard	0.1260697	0.245
	_cons	0.7520317	0.273

Coef., regression coefficient; WMH, white matter hyperintensity.

Analysis evaluates the impact of algorithm type, scanning parameters, external validation, software, field strength, multicenter status, and country on segmentation performance. Statistical significance was set at *p* < 0.05.

#### 3.4.4 Sensitivity analysis

Results of the leave-one-out sensitivity analysis are depicted in Supplementary Figure S1. Exclusion of any single study did not materially alter the pooled DSC, indicating that the findings were robust.

#### 3.4.5 Assessment of publication Bias

Findings from the publication bias assessment are summarized in Table 4 and illustrated in Figure 5. Neither the Begg rank correlation test (*Z* = 1.28, *p* = 0.200) nor the Egger linear regression test (*t* = −2.19, *p* = 0.051) yielded a statistically significant result. Moreover, visual inspection revealed the funnel plot to be largely symmetrical. Collectively, these findings do not support the presence of significant publication bias.

**Table 4:** Publication bias analysis for white matter hyperintensity, cerebral small vessel disease, and Alzheimer’s disease segmentation using Begg’s and Egger’s tests.

Brain region	Begg’s test		Egger’s test	
	*Z*	*p*	*t*	*p*
WMH	1.28	0.200	−2.19	0.051
CSVD	0.24	0.806	−4.826912	0.545
AD	1.11	0.266	−6.603302	0.341

WMH, white matter hyperintensity; CSVD, cerebral small vessel disease; AD, Alzheimer’s disease.

**Figure 5:**
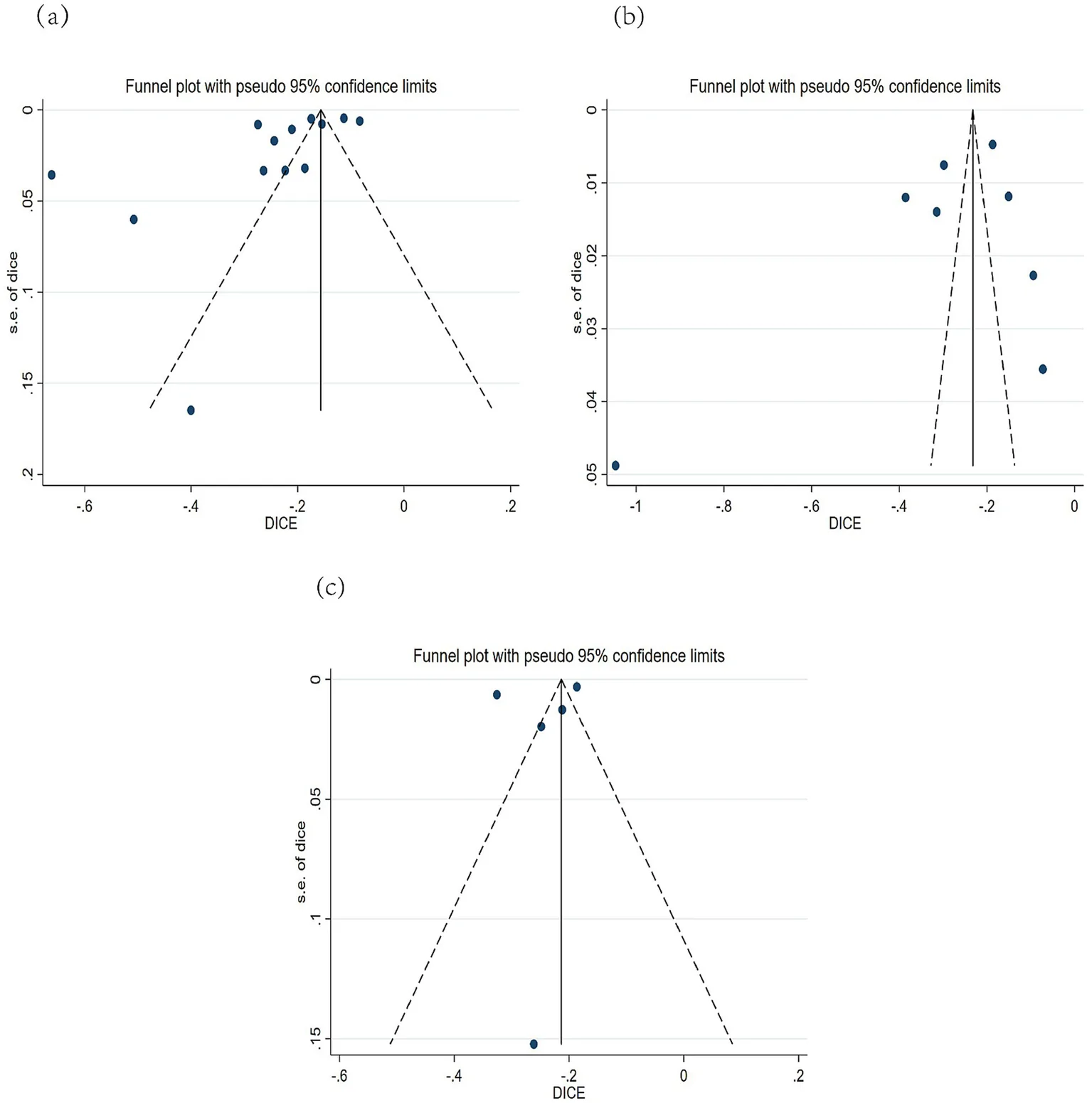
Funnel plots assessing publication bias in the meta-analyses of general/mixed WMH segmentation, Alzheimer’s disease-specific WMH segmentation, and cerebral small vessel disease-specific WMH segmentation. Funnel plots display effect sizes against standard errors for **(a)** white matter hyperintensity (WMH), **(b)** Alzheimer’s disease (AD), and **(c)** cerebral small vessel disease (CSVD).

## 4 Discussion

This systematic review and meta-analysis provide a comprehensive evaluation of AI algorithms for WMH segmentation across different disease contexts. The results indicate that AI achieves moderate-to-high segmentation performance, with a pooled DSC of 0.78. However, substantial heterogeneity was observed across studies, suggesting considerable variability in model performance. Exploratory univariable meta-regression suggested an association between scanning parameters and reported DSC values, while the association with magnetic field strength was borderline. These findings are hypothesis-generating and do not establish independent or causal effects. These exploratory findings suggest that differences in imaging protocols may contribute to variability in reported DSC values, but their independent effects remain uncertain.

The associations observed between DSC and scanning parameters, as well as the borderline association with magnetic field strength, should be interpreted as exploratory. The number of studies available for meta-regression was limited, and covariate information was incompletely reported for some studies. In addition, imaging characteristics, participant populations, lesion burden, reference-standard procedures, and model-development practices are correlated across studies. Therefore, the present analyses cannot establish an independent or causal effect of scanning parameters or field strength on segmentation performance.

To provide context rather than a benchmark of equivalence, Heinen et al. (2019) reported an inter-rater DSC of 0.80 ± 0.09 for a subset of reference segmentations generated using a semi-automated iso-contouring procedure. Although this value is numerically close to the pooled AI DSC (0.78), the different populations and annotation workflows preclude a claim of human-equivalent performance. Future work should therefore focus on generalisability, robustness, and clinical utility.

U-Net architectures and their variants had the highest descriptive pooled DSC. Because the studies used different datasets, reference standards, and validation designs, this pattern should not be interpreted as evidence of algorithmic superiority (Kuijf et al., 2019). Emerging approaches, including transformer-based models, foundation models, domain adaptation, self-supervised learning, and federated learning, require standardized multicentre external validation for WMH segmentation.

Disease-specific analyses yielded a numerically higher pooled DSC in CSVD-specific studies (0.78) than in AD-specific studies (0.75). This difference should not be interpreted as evidence of superior algorithmic performance in CSVD. DSC is sensitive to lesion burden and morphology: when WMH lesions are smaller, sparser, or more diffuse, a similar absolute number of discordant voxels can result in a larger proportional reduction in DSC. Differences in lesion burden, lesion distribution, MRI protocols, reference-label construction, dataset composition, and validation strategies may therefore have contributed to the observed variation between AD and CSVD subgroups. Because lesion-volume and lesion-morphology data were incompletely reported, these factors could not be assessed quantitatively. Accordingly, the disease-specific pooled estimates should be interpreted as descriptive summaries rather than evidence of disease-specific differences in algorithmic accuracy.

Interpretation of DSC requires consideration of the study-specific reference standards. Across the included studies, reference masks were based on manual delineation (single-rater or consensus), semi-automated segmentation with manual editing, or incompletely reported procedures. We retained DSC because it was the most consistently reported overlap metric; however, the pooled value reflects agreement with heterogeneous study-specific reference masks rather than objective biological ground truth. More broadly, the choice and interpretation of segmentation metrics should be aligned with the scientific or clinical task, because overlap-based metrics alone do not capture all clinically relevant error modes (Reinke et al., 2024; Maier-Hein et al., 2024).

The exploratory reference-standard analysis was limited by sparse categories and incomplete reporting; a non-significant association cannot exclude an effect on DSC or heterogeneity. Future studies should report the number and expertise of annotators, consensus procedures, manual editing, and inter-rater agreement.

AI-based WMH segmentation may support large-scale imaging research and longitudinal quantification, but the current evidence does not establish improved risk stratification or clinical decision-making. Prospective multicentre validation, standardized protocols, and evaluation of clinical impact are needed before routine implementation.

This review used a systematic search and a unified meta-analytic framework, but the exploratory meta-regression and publication-bias assessment should be interpreted cautiously given the small number of studies and substantial heterogeneity.

Several limitations should be acknowledged. The extreme heterogeneity, small subgroup sizes, and incomplete reporting of MRI protocols, reference standards, lesion burden, and external validation limit the interpretation of the pooled DSC. DSC captures overlap with a study-specific reference mask but not boundary error, lesion-wise detection, or volumetric agreement; complementary metrics were too inconsistently reported for quantitative synthesis. In addition, QUADAS-2 does not fully address several AI-specific risks, including data leakage, model-selection bias, limited reproducibility, and dataset shift. Accordingly, the findings should be interpreted cautiously. Future studies should follow AI-specific reporting guidance, such as CLAIM (Mongan et al., 2020), and use validation designs that minimize optimism from model selection and quantify performance in independent external datasets (Bradshaw et al., 2023).

## 5 Conclusion

In conclusion, AI-based WMH segmentation shows promise for research applications and longitudinal quantification, with U-Net–based approaches yielding the highest descriptive pooled DSC. However, the substantial between-study heterogeneity, heterogeneous reference standards, and limited multicentre external validation restrict the clinical interpretation and generalisability of the findings. Prospective multicentre studies using standardized imaging and annotation protocols, rigorous external validation, and direct evaluation of clinical impact are required before routine clinical implementation can be recommended.

## Data Availability

The original contributions presented in the study are included in the article/Supplementary material, further inquiries can be directed to the corresponding authors.

## References

[ref1] Bradshaw T. J. Huemann Z. Hu J. Rahmim A. (2023). A guide to cross-validation for artificial intelligence in medical imaging. Radiol. Artif. Intell. 5:e220232. doi: 10.1148/ryai.220232, 37529208 PMC10388213

[ref2] Cheng Z. Li M. Li J. Zhang N. Che Y. Chen Y. . (2025). Long-term impact of white matter hyperintensities and amyloid beta on thalamic subregions in cerebral small vessel disease: a prospective cohort study. Alzheimers Dement. 21:e70553. doi: 10.1002/alz.70553, 40819213 PMC12358008

[ref3] Duering M. Biessels G. J. Brodtmann A. Chen C. Cordonnier C. de Leeuw F. E. . (2023). Neuroimaging standards for research into small vessel disease-advances since 2013. Lancet Neurol. 22, 602–618. doi: 10.1016/S1474-4422(23)00131-X, 37236211

[ref4] Eldianto M. N. D. Rachmadi M. F. Jatmiko W. (2023). “White matter Hyperintensities segmentation using probabilistic TransUNet,” in 2023 International Conference on Computer Science, Information Technology and Engineering (ICCoSITE). doi: 10.1109/ICCoSITE57641.2023.10127707

[ref5] Fiford C. M. Sudre C. H. Pemberton H. Walsh P. Manning E. Malone I. B. . (2020). Automated white matter hyperintensity segmentation using Bayesian model selection: assessment and correlations with cognitive change. Neuroinformatics 18, 429–449. doi: 10.1007/s12021-019-09439-6, 32062817 PMC7338814

[ref6] He H. Jiang J. Peng S. He C. Sun T. Fan F. . (2025). A robust automated segmentation method for white matter hyperintensity of vascular-origin. NeuroImage 315:121279. doi: 10.1016/j.neuroimage.2025.121279, 40389145

[ref7] Heinen R. Steenwijk M. D. Barkhof F. Biesbroek J. M. van der Flier W. M. Kuijf H. J. . (2019). Performance of five automated white matter hyperintensity segmentation methods in a multicenter dataset. Sci. Rep. 9:16742. doi: 10.1038/s41598-019-52966-0, 31727919 PMC6856351

[ref8] Hotz I. Deschwanden P. F. Liem F. Mérillat S. Malagurski B. Kollias S. . (2022). Performance of three freely available methods for extracting white matter hyperintensities: FreeSurfer, UBO detector, and BIANCA. Hum. Brain Mapp. 43, 1481–1500. doi: 10.1002/hbm.25739, 34873789 PMC8886667

[ref9] Huang F. Xia P. Vardhanabhuti V. Hui S. K. Lau K. K. Ka-Fung Mak H. . (2023). Semisupervised white matter hyperintensities segmentation on MRI. Hum. Brain Mapp. 44, 1344–1358. doi: 10.1002/hbm.26109, 36214210 PMC9921214

[ref10] Ilesanmi A. E. Ilesanmi T. O. Ajayi B. O. (2024). Reviewing 3D convolutional neural network approaches for medical image segmentation. Heliyon 10:e27398. doi: 10.1016/j.heliyon.2024.e27398, 38496891 PMC10944240

[ref11] Iveson M. H. Mukherjee M. Davidson E. M. Zhang H. Sherlock L. Ball E. L. . (2026). Clinically reported covert cerebrovascular disease and risk of neurological disease: a whole-population cohort of 367 988 people using natural language processing. J. Neurol. Neurosurg. Psychiatry 97, 630–638. doi: 10.1136/jnnp-2025-337689, 41825869 PMC13288941

[ref12] Jansma A. de Bresser J. Schoones J. W. van Heemst D. Akintola A. A. (2024). Sporadic cerebral small vessel disease and cognitive decline in healthy older adults: a systematic review and meta-analysis. J. Cereb. Blood Flow Metab. 44, 660–679. doi: 10.1177/0271678X241235494, 38415688 PMC11197143

[ref13] Jeong S. Y. Jung W. Suh C. H. Kim S. Y. Kim J. Heo H. . (2025). Development and validation of a deep learning-based automatic segmentation and classification of cerebral white matter hyperintensities. Eur. Radiol. 36, 4812–4823. doi: 10.1007/s00330-025-12174-z, 41398110

[ref14] Johnsen L. H. Herder M. Vangberg T. R. Isaksen J. G. Mathiesen E. B. (2025). Association between intracerebral artery stenosis and cortical infarcts, lacunes, white matter hyperintensities and brain atrophy: results from a population-based study. Cerebrovasc. Dis. Extra 15, 225–232. doi: 10.1159/000548650, 41078171 PMC12659642

[ref15] Kervadec H. Bouchtiba J. Desrosiers C. Granger E. Dolz J. Ben Ayed I. (2021). Boundary loss for highly unbalanced segmentation. Med. Image Anal. 67:101851. doi: 10.1016/j.media.2020.101851, 33080507

[ref16] Kuijf H. J. Casamitjana A. Collins D. L. Dadar M. Georgiou A. Ghafoorian M. . (2019). Standardized assessment of automatic segmentation of white matter hyperintensities and results of the WMH segmentation challenge. IEEE Trans. Med. Imaging 38, 2556–2568. doi: 10.1109/TMI.2019.2905770, 30908194 PMC7590957

[ref17] Li X. Zhao Y. Jiang J. Cheng J. Zhu W. Wu Z. . (2022). White matter hyperintensities segmentation using an ensemble of neural networks. Hum. Brain Mapp. 43, 929–939. doi: 10.1002/hbm.25695, 34704337 PMC8764480

[ref18] Liang L. Zhou P. Lu W. Guo X. Ye C. Lv H. . (2021). An anatomical knowledge-based MRI deep learning pipeline for white matter hyperintensity quantification associated with cognitive impairment. Comput. Med. Imaging Graph. 89:101873. doi: 10.1016/j.compmedimag.2021.101873, 33610084

[ref19] Lo L. T. Tsai Y-C Chang Y-J Ting H-W Chien T-Y. (2024). “Constructing a segmentation model for patients with Leukoaraiosis using deep learning algorithms,” in ACM International Conference Proceeding Series, (New York, NY, USA: Association for Computing Machinery).

[ref20] Maier-Hein L. Reinke A. Godau P. Tizabi M. D. Buettner F. Christodoulou E. . (2024). Metrics reloaded: recommendations for image analysis validation. Nat. Methods 21, 195–212. doi: 10.1038/s41592-023-02151-z, 38347141 PMC11182665

[ref21] McInnes M. D. F. Moher D. Thombs B. D. McGrath T. A. Bossuyt P. M. Clifford T. . (2018). Preferred reporting items for a systematic review and meta-analysis of diagnostic test accuracy studies: the PRISMA-DTA statement. JAMA 319, 388–396. doi: 10.1001/jama.2017.19163, 29362800

[ref22] Mojiri Forooshani P. Biparva M. Ntiri E. E. Ramirez J. Boone L. Holmes M. F. . (2022). Deep Bayesian networks for uncertainty estimation and adversarial resistance of white matter hyperintensity segmentation. Hum. Brain Mapp. 43, 2089–2108. doi: 10.1002/hbm.25784, 35088930 PMC8996363

[ref23] Mongan J. Moy L. Kahn C. E. Jr. (2020). Checklist for artificial intelligence in medical imaging (CLAIM): a guide for authors and reviewers. Radiol. Artif. Intell. 2:e200029. doi: 10.1148/ryai.2020200029, 33937821 PMC8017414

[ref24] Park J. H. Park K. I. Kim D. Lee M. Kang S. Kang S. J. . (2023). Improving performance robustness of subject-based brain segmentation software. Encephalitis 3, 24–33. doi: 10.47936/encephalitis.2022.00108, 37469714 PMC10295817

[ref25] Pham P. T. Tuan TA Le NLT Kim JY. (2021). “An adaptive 3D U-Net for white matter, gray matter and cerebrospinal fluid segmentation from 3D-brain MRI,” in ACM International Conference Proceeding Series, (New York, NY, USA: Association for Computing Machinery).

[ref26] Qin X. Deng C. Li X. Qin Z. Gong Y. (2026). MSA(2)-net: utilizing self-adaptive convolution module to extract multi-scale information in medical image segmentation. Sci. Prog. 109:368504261432413. doi: 10.1177/00368504261432413, 41940578 PMC13053973

[ref27] Qiu Y. Hu Y. Ding W. Fu Q. Hu W. Wang Y. . (2025). White matter hyperintensity-associated iron overload links glymphatic system dysfunction to cognitive impairment in cerebral small vessel disease. NeuroImage 321:121515. doi: 10.1016/j.neuroimage.2025.121515, 41072636

[ref28] Rachmadi M. F. Valdés-Hernández M. C. Agan M. L. F. di Perri C. Komura T. (2018). Segmentation of white matter hyperintensities using convolutional neural networks with global spatial information in routine clinical brain MRI with none or mild vascular pathology. Comput. Med. Imaging Graph. 66, 28–43. doi: 10.1016/j.compmedimag.2018.02.002, 29523002

[ref29] Ramirez J. Gibson E. Quddus A. Lobaugh N. J. Feinstein A. Levine B. . (2011). Lesion explorer: a comprehensive segmentation and parcellation package to obtain regional volumetrics for subcortical hyperintensities and intracranial tissue. NeuroImage 54, 963–973. doi: 10.1016/j.neuroimage.2010.09.013, 20849961

[ref30] Reinke A. Tizabi M. D. Baumgartner M. Eisenmann M. Heckmann-Nötzel D. Kavur A. E. . (2024). Understanding metric-related pitfalls in image analysis validation. Nat. Methods 21, 182–194. doi: 10.1038/s41592-023-02150-0, 38347140 PMC11181963

[ref31] Rieu Z. Kim J. Y. Kim R. E. Y. Lee M. Lee M. K. Oh S. W. . (2021). Semi-supervised learning in medical MRI segmentation: brain tissue with white matter hyperintensity segmentation using FLAIR MRI. Brain Sci. 11:720. doi: 10.3390/brainsci11060720, 34071634 PMC8228966

[ref32] Røvang M. S. Selnes P. MacIntosh B. J. Rasmus Groote I. Pålhaugen L. Sudre C. . (2023). Segmenting white matter hyperintensities on isotropic three-dimensional fluid attenuated inversion recovery magnetic resonance images: assessing deep learning tools on a Norwegian imaging database. PLoS One 18:e0285683. doi: 10.1371/journal.pone.0285683, 37616243 PMC10449185

[ref33] Shan W. Duan Y. Zheng Y. Wu Z. Chan S. W. Wang Q. . (2021). Segmentation of cerebral small vessel diseases-white matter hyperintensities based on a deep learning system. Front. Med. (Lausanne) 8:681183. doi: 10.3389/fmed.2021.681183, 34901045 PMC8656685

[ref34] Sundaresan V. Dinsdale N. K. Jenkinson M. Griffanti L. (2022). “Omni-supervised domain adversarial training for white matter hyperintensity segmentation in the UK biobank,” in 2022 IEEE 19th International Symposium on Biomedical Imaging (ISBI). Oxford.

[ref35] Tsuchida A. Boutinaud P. Verrecchia V. Tzourio C. Debette S. Joliot M. (2024). Early detection of white matter hyperintensities using SHIVA-WMH detector. Hum. Brain Mapp. 45:e26548. doi: 10.1002/hbm.26548, 38050769 PMC10789222

[ref36] Vanderbecq Q. Xu E. Ströer S. Couvy-Duchesne B. Diaz Melo M. Dormont D. . (2020). Comparison and validation of seven white matter hyperintensities segmentation software in elderly patients. Neuroimage Clin. 27:102357. doi: 10.1016/j.nicl.2020.102357, 32739882 PMC7394967

[ref37] Viteri J. A. Piguave B. Pelaez C. E. Loayza F. (2022). “Automatic brain white matter hyperintensities segmentation with Swin U-Net,” in 2022 IEEE ANDESCON. IEEE.

[ref38] Wardlaw J. M. Smith E. E. Biessels G. J. Cordonnier C. Fazekas F. Frayne R. . (2013). Neuroimaging standards for research into small vessel disease and its contribution to ageing and neurodegeneration. Lancet Neurol. 12, 822–838. doi: 10.1016/S1474-4422(13)70124-8, 23867200 PMC3714437

[ref39] Whitman G. T. Tang T. Lin A. Baloh R. W. (2001). A prospective study of cerebral white matter abnormalities in older people with gait dysfunction. Neurology 57, 990–994. doi: 10.1212/WNL.57.6.990, 11571322

[ref40] WuJ. BrownJ. D. HuR. EdwardsP. J. LeveyA. I. LahJ. J. . (2026). Benchmark white matter Hyperintensity segmentation methods fail on heterogeneous clinical MRI: a new dataset and deep learning-based solutions. J. Imaging Inform. Med. doi: 10.1007/s10278-025-01808-9 (online ahead of print)., 41612110

[ref41] Wu J. Zhang Y. Wang K. Tang X. (2019). Skip connection U-Net for white matter hyperintensities segmentation from MRI. IEEE Access 7, 155194–155202. doi: 10.1109/access.2019.2948476

[ref42] Zhang Z. Wu C. Coleman S. Kerr D. (2020). DENSE-INception U-Net for medical image segmentation. Comput. Methods Prog. Biomed. 192:105395. doi: 10.1016/j.cmpb.2020.105395, 32163817

[ref43] Zhang Y. Chen W. Chen Y. Tang X. (2018). “A post-processing method to improve the white matter hyperintensity segmentation accuracy for randomly-initialized U-Net,” in 2018 IEEE 23rd International Conference on Digital Signal Processing (DSP).

[ref44] Zhang F. Wang Y. Wu L. Liu M. Hu S. Li M. (2021). “GLM-net: a multi-scale image segmentation network for brain abnormalities based on GLCM,” in 2021 14th International Congress on Image and Signal Processing, BioMedical Engineering and Informatics. (IEEE DSP: CISP-BMEI).

[ref45] Zhao X. Wang X. Wang H. (2021). “PRU-Net: an U-Net model with pyramid pooling and residual block for WMH segmentation,” in Communications in Computer and Information Science, (IEEE CISP-BMEI, IEEE. Singapore: Springer Science and Business Media Deutschland GmbH).

[ref46] Zhao H. Zhang M. Tang W. Jin L. Tang J. Shi L. . (2025). Deep learning-based automated segmentation for the quantitative diagnosis of cerebral small vessel disease via multisequence MRI. Front. Neurol. 16:1540923. doi: 10.3389/fneur.2025.1540923, 40496122 PMC12150800

