# Supplementary material for: Artificial intelligence for white matter hyperintensity segmentation: performance, determinants, and clinical implications
Source: Front Aging Neurosci. 2026 Sep 16;18:1930675. doi: 10.3389/fnagi.2026.1930675 (PMC13624168; doi:10.3389/fnagi.2026.1930675)
Supplement: Supplementary file 1 [file Data_Sheet_1.docx]

**Pubmed**

((("White Matter"[Mesh]) OR (((((((((Matters, White) OR (Matter, White)) OR (White Matters)) OR (Cerebellar White Matter)) OR (Cerebellar White Matters)) OR (Matter, Cerebellar White)) OR (Matters, Cerebellar White)) OR (White Matter, Cerebellar)) OR (White Matters, Cerebellar))) AND (((("Artificial Intelligence"[Mesh]) OR (((((((((((((((((((ntelligence, Artificial) OR (Computer Reasoning)) OR (Reasoning, Computer)) OR (AI (Artificial Intelligence))) OR (Machine Intelligence)) OR (Intelligence, Machine)) OR (Computational Intelligence)) OR (Intelligence, Computational)) OR (Computer Vision Systems)) OR (Computer Vision System)) OR (System, Computer Vision)) OR (Systems, Computer Vision)) OR (Vision System, Computer)) OR (Vision Systems, Computer)) OR (Knowledge Acquisition (Computer))) OR (Acquisition, Knowledge (Computer))) OR (Knowledge Representation (Computer))) OR (Knowledge Representations (Computer))) OR (Representation, Knowledge (Computer)))) OR (("Machine Learning"[Mesh]) OR (((Learning, Machine) OR (Transfer Learning)) OR (Learning, Transfer)))) OR (("Deep Learning"[Mesh]) OR (((Learning, Deep) OR (Hierarchical Learning)) OR (Learning, Hierarchical))))) AND ((segmentation) OR (Segmentation))

**IEEE**

(("All Metadata":White Matter) AND("All Metadata":segmentation)AND(("All Metadata":Artificial Intelligence) OR ("All Metadata":Machine Learning) OR ("All Metadata":Deep Learning)))

**Scopus**

( ( TITLE-ABS-KEY ( White Matter ) OR TITLE-ABS-KEY ( Matters , White. ) OR TITLE-ABS-KEY ( Matter , White ) OR TITLE-ABS-KEY ( White Matters ) OR TITLE-ABS-KEY ( Cerebellar White Matter ) OR TITLE-ABS-KEY ( Cerebellar White Matters ) OR TITLE-ABS-KEY ( Matter , Cerebellar White ) OR TITLE-ABS-KEY ( Matters , Cerebellar White ) OR TITLE-ABS-KEY ( White Matter , Cerebellar ) OR TITLE-ABS-KEY ( White Matters , Cerebellar ) ) ) AND ( ( TITLE-ABS-KEY ( image segmentation ) OR TITLE-ABS-KEY ( images segmentations ) OR TITLE-ABS-KEY ( segmentation methods ) OR TITLE-ABS-KEY ( segmentation algorithms ) OR TITLE-ABS-KEY ( segmentation ) ) ) AND ( ( ( TITLE-ABS-KEY ( Artificial Intelligence ) OR TITLE-ABS-KEY ( ntelligence , Artificial ) OR TITLE-ABS-KEY ( Computer Reasoning ) OR TITLE-ABS-KEY ( Reasoning , Computer ) OR TITLE-ABS-KEY ( AI ) OR TITLE-ABS-KEY ( Machine Intelligence ) OR TITLE-ABS-KEY ( Intelligence , Machine ) OR TITLE-ABS-KEY ( Computational Intelligence ) OR TITLE-ABS-KEY ( Intelligence , Computational ) OR TITLE-ABS-KEY ( Computer Vision Systems ) OR TITLE-ABS-KEY ( Computer Vision System ) OR TITLE-ABS-KEY ( System , Computer Vision ) OR TITLE-ABS-KEY ( Systems , Computer Vision ) OR TITLE-ABS-KEY ( Vision System , Computer ) OR TITLE-ABS-KEY ( Vision Systems , Computer ) OR TITLE-ABS-KEY ( Knowledge Acquisition ) OR TITLE-ABS-KEY ( Acquisition , Knowledge ) OR TITLE-ABS-KEY ( Knowledge Representation ) OR TITLE-ABS-KEY ( Knowledge Representations ) OR TITLE-ABS-KEY ( Representation , Knowledge ) ) ) OR ( ( TITLE-ABS-KEY ( Machine Learning ) OR TITLE-ABS-KEY ( Learning , Machine ) OR TITLE-ABS-KEY ( Transfer Learning ) OR TITLE-ABS-KEY ( Learning , Transfer ) ) ) OR ( ( TITLE-ABS-KEY ( Deep Learning ) OR TITLE-ABS-KEY ( Learning , Deep ) OR TITLE-ABS-KEY ( Hierarchical Learning ) OR TITLE-ABS-KEY ( Learning , Hierarchical ) ) ) )

**Web of science**

(White Matter (Topic) or Matters, White. (Topic) or Matter, White (Topic) or White Matters (Topic) or Cerebellar White Matter (Topic) or Cerebellar White Matters (Topic) or Matter, Cerebellar White (Topic) or Matters, Cerebellar White (Topic) or White Matter, Cerebellar (Topic) or White Matters, Cerebellar (Topic))AND(Artificial Intelligence (Topic) or intelligence, Artificial (Topic) or Computer Reasoning (Topic) or Reasoning, Computer (Topic) or AI (Artificial Intelligence) (Topic) or Machine Intelligence (Topic) or Intelligence, Machine (Topic) or Computational Intelligence (Topic) or Intelligence, Computational (Topic) or Computer Vision Systems (Topic) or Computer Vision System (Topic) or System, Computer Vision (Topic) or Systems, Computer Vision (Topic) or Vision System, Computer (Topic) or Vision Systems, Computer (Topic) or Knowledge Acquisition (Computer) (Topic) or Acquisition, Knowledge (Computer) (Topic) or Knowledge Representation (Computer) (Topic) or Knowledge Representations (Computer) (Topic) or Representation, Knowledge (Computer) (Topic)) AND ((Machine Learning (Topic) or Learning, Machine (Topic) or Transfer Learning (Topic) or Learning, Transfer (Topic))OR(Deep Learning (Topic) or Learning, Deep (Topic) or Hierarchical Learning (Topic) or Learning, Hierarchical (Topic)) AND(segmentation (Topic) or Segmentation (Topic))

**Embase**

('white matter'/exp OR 'brain white matter' OR 'cerebellar white matter' OR 'cerebellum white matter' OR 'cerebral white matter' OR 'spinal cord white matter' OR 'spinal white matter' OR 'substantia alba' OR 'substantia alba cerebelli' OR 'substantia alba medullae spinalis' OR 'white matter')AND ('cognition disorder' OR 'cognition disorders' OR 'cognitive complaints' OR 'cognitive decline' OR 'cognitive defects' OR 'cognitive deficiency' OR 'cognitive deficit' OR 'cognitive difficulties' OR 'cognitive disability' OR 'cognitive disorder' OR 'cognitive disorders' OR 'cognitive disturbance' OR 'cognitive dysfunction' OR 'cognitive impairment' OR 'cognitive problems' OR 'delirium, dementia, amnestic, cognitive disorders' OR 'overinclusion' OR 'response interference' OR 'cognitive defect') AND (segmentation)

**Table S1 QUADAS‑2 quality assessment of the included studies**

| **Study** | **Year** | **Patient Selection** | | **Index Test** | | **Reference Standard** | | **Flow and Timing** | |
| --- | --- | --- | --- | --- | --- | --- | --- | --- | --- |
|  |  | **Risk of Bias** | **Applicability** | **Risk of Bias** | **Applicability** | **Risk of Bias** | **Applicability** | **Risk of Bias** | **Applicability** |
| Zhao | 2021 | Low | Low | Unclear | Low | Low | Low | Low | Low |
| Zhang | 2018 | Low | Low | Low | Low | Low | Low | Low | Low |
| Zhang(b) | 2021 | Low | Low | Low | Low | Low | Low | Low | Low |
| WU | 2017 | Low | Low | Low | Low | Low | Low | Low | Low |
| WU(b) | 2026 | Low | Low | Low | Low | Low | Low | Low | Low |
| Viteri | 2022 | Low | Low | Low | Low | Low | Low | Low | Low |
| Tsuchida | 2023 | Low | Low | Low | Low | Low | Low | Low | Low |
| Sundaresan | 2022 | Low | Low | Low | Low | Low | Low | Low | Low |
| Rieu | 2021 | Low | Low | Low | Low | Low | Low | Low | Low |
| Rachmadi | 2018 | Low | Low | Low | Low | Low | Low | Low | Low |
| Forooshani | 2021 | Low | Low | Low | Low | Low | Low | Low | Low |
| Lo | 2024 | Low | Low | Low | Low | Low | Low | Low | Low |
| Hotz | 2022 | Low | Low | Low | Low | Low | Low | Low | Low |
| Zhao(b) | 2025 | Low | Low | Low | Low | Low | Low | Low | Low |
| Liang | 2021 | Low | Low | Low | Low | Low | Low | Low | Low |
| Shan | 2021 | Low | Low | Low | Low | Low | Low | Low | Low |
| Li | 2022 | Low | Low | Low | Low | Low | Low | Low | Low |
| Jeong | 2025 | Low | Low | Low | Low | Low | Low | Low | Low |
| Huang | 2022 | Low | Low | Low | Low | Low | Low | Low | Low |
| He | 2025 | Low | Low | Low | Low | Low | Low | Low | Low |
| Fiford | 2020 | Low | Low | Low | Low | Low | Low | Low | Low |
| Eldianto | 2023 | Low | Low | Low | Low | Low | Low | Low | Low |
| Vanderbecq | 2020 | Low | Low | Low | Low | Low | Low | Low | Low |
| Røvang | 2023 | Low | Low | Low | Low | Low | Low | Low | Low |
| Pham | 2021 | Low | Low | Low | Low | Low | Low | Low | Low |
| Park | 2022 | Low | Low | Low | Low | Low | Low | Low | Low |

The Quality Assessment of Diagnostic Accuracy Studies (QUADAS‑2) tool was used to evaluate the risk of bias and applicability concerns across four domains: patient selection, index test, reference standard, and flow and timing. Each study was rated as “low,” “high,” or “unclear” for each domain. All included studies were rated as low risk of bias and low concern regarding applicability across all domains, indicating overall high methodological quality.

**Table S2. Reference-standard characteristics of included studies**

| Study | Year | Reference-standard |
| --- | --- | --- |
| Zhao | 2021 | Multi-rater consensus |
| Zhang | 2018 | Multi-rater consensus |
| Zhang(b) | 2021 | Not reported |
| WU | 2017 | Multi-rater consensus |
| WU(b) | 2026 | Multi-rater consensus |
| Viteri | 2022 | Multi-rater consensus |
| Tsuchida | 2023 | Not reported |
| Sundaresan | 2022 | Not reported |
| Rieu | 2021 | Multi-rater consensus |
| Rachmadi | 2018 | Semi-automated + manual editing |
| Forooshani | 2021 | Semi-automated + manual editing |
| Lo | 2024 | Not reported |
| Hotz | 2022 | Multi-rater consensus |
| Zhao(b) | 2025 | Multi-rater consensus |
| Shan | 2021 | Not reported |
| Li | 2022 | Multi-rater consensus |
| Jeong | 2025 | Not reported |
| Huang | 2022 | Multi-rater consensus |
| He | 2025 | Multi-rater consensus |
| Fiford | 2020 | Semi-automated + manual editing |
| Eldianto | 2023 | Not reported |
| Vanderbecq | 2020 | Single-rater manual |
| Røvang | 2023 | Semi-automated + manual editing |
| Pham | 2021 | Not reported |
| Park | 2022 | Not reported |
| Liang | 2021 | Multi-rater consensus |

WMH, white matter hyperintensity; NR, not reported. Reference-standard construction was classified as manual annotation, semi-automated segmentation followed by manual editing, or insufficiently reported. No included study used an automated algorithm alone as the reference standard.

Figure S1 Figure 4 Sensitivity analysis of the combined Dice similarity coefficient of white matter hyperintensity segmentation by disease type.

Panels (a) and (b) present sensitivity analyses for Alzheimer’s disease (AD) and cerebral small vessel disease (CSVD), respectively. Sensitivity analysis was performed using the leave‑one‑out method to assess the influence of individual studies on the pooled Dice similarity coefficient (DSC). The results demonstrate that no single study disproportionately influenced the overall effect size, confirming the robustness of the meta‑analysis findings.
